# Degree of dependence influences the effect of smoking on cognitive flexibility

**DOI:** 10.1016/j.pbb.2011.01.015

**Published:** 2011-05

**Authors:** J. Nesic, J. Rusted, T. Duka, A. Jackson

**Affiliations:** a Department of Pharmacology and Therapeutics, School of Pharmacy and Biomolecular Sciences, University of Brighton, Moulsecoomb, Brighton BN2 4GJ, UK; b School of Psychology, University of Sussex, Falmer, Brighton BN1 9QG, UK

**Keywords:** Nicotine, Dependence, Cotinine, Fagerström Tolerance Questionnaire (FTQ), Cognitive flexibility, Intra-Extra Dimensional Set-Shift (IED) test

## Abstract

Pre-frontal cortical (PFC) dysfunction has been put forward as the basis for development and maintenance of addiction. To explore this relationship, the present study investigated the effects of smoking on PFC-mediated cognitive flexibility and subjective states in low- (LD) and high-dependent (HD) smokers.

Twenty-four LD and 24 HD smokers (Fagerström dependence scores ≤ 4 and ≥ 5, respectively) were randomly allocated to non-smoking or smoking condition (12 LD and 12 HD participants per condition). After abstaining from smoking for a minimum of two hours volunteers completed a battery of questionnaires [nicotine-specific Visual Analogue Scales (Nic-VAS), Questionnaire of Smoking Urges (QSU) and Profile of Mood States (POMS)] at baseline [T1] and again after smoking one cigarette or remaining abstinent [T2]. Cognitive flexibility was evaluated at T2 using the Intra-Extra Dimensional Set-Shift test. The Rapid Visual Information Processing test was performed as a control nicotine-sensitive task at several time points during the experiment.

Compared to LD smokers, HD smokers had higher salivary cotinine and breath CO levels at baseline and reported more craving (QSU) and felt less stimulated (Nic-VAS), vigorous, friendly and elated (POMS) throughout the experiment. Smoking increased Nic-VAS ratings of ‘Buzzed’ and ‘Dizzy’ and decreased craving in all participants. Smoking selectively impaired cognitive flexibility in HD smokers since HD smokers allocated to the smoking condition made significantly more errors with the intra-dimensional set-shift than their counterparts in the abstinent condition. No effect of smoking on RVIP test was observed, most likely due to the practice effect which was significant in both groups of smokers. The practice effect, however, was more pronounced in LD smokers.

This study demonstrates that PFC-mediated cognitive effects of smoking as well as subjective reports vary according to the degree of nicotine dependence.

## Research Highlights

► High-, compared to low-dependent smokers feel less stimulated, vigorous, friendly and elated. ► High dependent smokers report more craving for cigarettes. ► Smoking induces subjective effects and reduced craving in all participants. ► Smoking selectively impairs cognitive flexibility in high dependent smokers.

## 1 Introduction

Pre-frontal cortical (PFC) dysfunction has been put forward as the basis for development and maintenance of drug addiction (Kalivas and Volkow, 2005). According to Goldstein and Volkow (2002), drug addiction can be defined as ‘a syndrome of impaired response inhibition and salience attribution’ (p. 1643), and is thought to stem from the reduced inhibitory control of behavior by pre-frontal structures, which accentuates behavior driven by motivationally significant stimuli. In support of this view, abstinent drug abusers were found to display impairments in several components of frontal executive cognitive function compared to healthy controls (Verdejo-Garcia et al., 2004, 2005, 2006; Kim et al., 2005; von Geusau et al., 2004; Ornstein et al., 2000; Paul et al., 2006), as well as to show reduced glucose utilization in the PFC, which was correlated with the extent of the cognitive impairment (Kim et al., 2005). These findings, however, are not unequivocal and a few studies reported no difference (Verdejo-Garcia and Pérez-Garcia, 2007; Johanson et al., 2006) or even better performance (Hoff et al., 1996) of substance abusers relative to healthy controls in tests of executive function.

While abusers of cannabis, stimulants and heroin have been shown to suffer from impairments in frontal executive function, as exhibited by reduced cognitive flexibility (Lundqvist, 2005; Ornstein et al., 2000; Verdejo-Garcia et al., 2005), very little data exists regarding cognitive flexibility in smokers. Reports of a negative relationship between the severity of smoking dependence and cognitive flexibility in adolescent psychiatric patients (Martin et al., 2000) as well as in healthy middle-aged individuals (Kalmijn et al., 2002) suggest that pre-frontal dysfunction may also be implicated in nicotine abuse. The findings from both of these studies (Martin et al., 2000; Kalmijn et al., 2002), however, were only correlational and smoking satiation was not experimentally manipulated — subjects were tested in a no-smoking environment. It is therefore possible that the observed cognitive impairments were due to nicotine withdrawal and that the heavier smokers had the greatest impairments due to the greatest severity of withdrawal. On the other hand, in a controlled abstinence study, Rotheram-Fuller et al. (2004) found no difference in cognitive flexibility between healthy non-smokers and smokers who were abstinent for 30 min. In addition, Mancuso et al. (1999) found no effect of nicotine patch on cognitive flexibility in heavy smokers who were abstinent for two hours prior to testing. The task used in the latter study, however, only measured reversal learning and not the more complex executive function that is involved in attentional set-shifting.

Although very few studies have examined the effects of smoking/nicotine administration on cognitive flexibility, the enhancing effects of nicotine have been successfully demonstrated in a number of studies of sustained attention and effortful or strategic cognitive processing (e.g. Bell et al., 1999; Domier et al., 2007; Warburton and Arnall, 1994; Le Houezec et al., 1994; Snyder and Henningfield, 1989; Tait et al., 2000; Foulds et al., 1996; Levin et al., 1998). While in most of these studies the positive cognitive effects of nicotine have been interpreted in terms of the reversal of withdrawal-induced deficits (Bell et al., 1999; Snyder and Henningfield, 1989; Tait et al., 2000; Domier et al., 2007), cognitive enhancement seen in minimally- or non-deprived smokers (Warburton and Arnall, 1994; Lawrence et al., 2002; Rycroft et al., 2005, 2006) as well as in non-smokers (Le Houezec et al., 1994; Foulds et al., 1996; Levin et al., 1998; Rusted and Trawley, 2006; Rusted and Alvares, 2008; Marchant et al., 2008; Holmes et al., 2008) suggests that nicotine does indeed facilitate some aspects of cognitive processing.

Smoking-induced cognitive enhancement, however, has not been consistently demonstrated across studies. For example, West and Hack (1991) reported smoking-induced improvement in performance on a complex information processing test (Sternberg memory search paradigm) both in occasional (mean 1.4 cigarettes per day) and in regular smokers (mean 14.6 cigarettes per day). In contrast, another study examining the performance of abstinent and non-abstinent smokers (≥ 20 cigarettes per day) on the Sternberg task found that both groups of smokers performed worse than the non-smokers (Spilich et al., 1992), with non-abstinent smokers performing the worst. The importance of the level of smoking dependence in determining the cognitive effects of smoking has further been demonstrated in a study by Tait et al. (2000), who observed a significant improvement in cognitive processing after smoking in previously abstinent heavy but not light smokers. Recently, Azizian et al. (2008) failed to demonstrate smoking-related improvement in perceptual–motor test performance which may have been due to the observed negative correlation between the severity of smoking dependence and the task performance across the two conditions (smoking and abstinent). Taken together, these studies suggest that the degree of dependence may be a factor that interacts with the experimental manipulation as to obscure the acute effect of smoking.

The outcome of cognitive studies is further complicated by the fact that the selection criteria used for participant selection in smoking research vary, with many studies focusing on number of cigarettes per day (e.g. Spilich et al., 1992; Tait et al., 2000; Mancuso et al., 1999). However, this is not always the most appropriate indicator of nicotine dependence (Etter and Perneger, 2001). For example, an individual may smoke 10–15 cigarettes per day and yet have a low dependence score because he or she has the first cigarette of the day several hours after waking and smokes mostly later in the day and in the evening, when socializing with friends. Conversely, another individual may smoke only 5–6 cigarettes per day and yet be in the habit of smoking first thing in the morning and regularly throughout the day. A more complex measure of dependence, the score derived from the Fagerström Tolerance Questionnaire (FTQ; Fagerström, 1978), incorporates other aspects of smoking behavior in addition to the number of cigarettes and thus may be more appropriate to detect the differences between low- and high-dependent smokers.

The aim of the present study was to investigate the acute effects of smoking on cognitive flexibility in low- and high-dependent smokers, as defined by the FTQ score. The test of pre-frontal executive function used in the present study was the Intra-Extra Dimensional Set Shifting task (IED), a computerized equivalent of the Wisconsin Card Sorting Test (WCST). The IED test consists of nine consecutive stages with increasing level of complexity and it measures two components of executive function: reversal learning (ability to inhibit a habitual response) and attentional flexibility (intra-dimensional and extra-dimensional set-shift stages). Different stages of the task have been found to promote activity in different regions of the PFC (Rogers et al., 2000), the extra-dimensional shift stage being the stage most sensitive to frontal lobe damage (Owen et al., 1991). In addition to the IED test, participants in this study also performed a Rapid Visual Information Processing (RVIP) test as a cognitive control task sensitive to nicotine effects (e.g. Warburton and Arnall, 1994; Foulds et al., 1996; Jackson et al., 2009).

## 2 Method

### 2.1 Subjects

Forty eight volunteers aged 18–35 (*mean ± SEM*: 23.3 ± 0.4) and smoking 1–30 cigarettes per day (*mean ± SEM*: 11.5 ± 1.1) were recruited from staff and students at Universities of Brighton and Sussex. They were fluent in English, generally healthy and not taking any medication at the time of the testing session. Volunteers were recruited on the basis of their smoking dependence score, as measured by the Fagerström Tolerance Questionnaire (FTQ; Fagerström, 1978), and allocated to either low-dependent (LD; FTQ score ≤ 4; 12 male, 12 female; 1–15 cigarettes per day) or high-dependent smoker group (HD; FTQ score ≥ 5; 12 male, 12 female; 5–30 cigarettes per day). The cut-off points for the two groups represent the 33rd and the 66th percentile FTQ score of a sample of 181 smokers who had completed the FTQ either for previous smoking studies at the University of Brighton or when joining the University of Brighton Psychopharmacology laboratory volunteer database. Because of a very small range of scores on the FTQ (minimum 1, maximum 11) and because the majority of smokers in our population had scores between 3 and 7, the 33rd percentile was 4, which was the same as the median, while the 66th percentile was 5. Half of each FTQ group was then randomly allocated to the smoking (S) condition and half to the abstinent (NS) condition (groups balanced for gender).

Volunteers were instructed to refrain from smoking for at least 2 h prior to the testing session and were told that compliance would be verified by administration of a Smokerlyzer test. They were asked to avoid using illicit drugs for at least one week, sleeping pills and other sedatives for 48 h and alcohol for 12 h before the testing session. Additionally, they were asked to abstain from consuming caffeinated drinks for one hour before the start of the testing session. At the beginning of the experiment volunteers received an information sheet which stated that the purpose of the study was to investigate the subjective and cognitive effects of smoking. They were informed that they may be asked to smoke one of their own cigarettes during the testing session. All volunteers gave their written informed consent to take part in the study and received £8 at the end of the testing session. This study was approved by the School of Pharmacy and Biomolecular Sciences Research Ethics Committee at the University of Brighton in compliance with the Declaration of Helsinki for the use of human participants.

### 2.2 Experimental design

Volunteers were tested individually in a between-subjects design, fully balanced for gender and the smoking dependence level, with the between-subjects factors being smoking condition (abstinent — NS vs. smoking — S) and FTQ group (low-dependent — LD vs. high-dependent — HD). In order to assess temporal changes during the course of the experimental session, ‘time point’ was introduced as a within-subject factor for some of the dependent variables. Dependent variables were subjective effects, mood, craving for cigarettes, breath CO and RVIP test performance (2 time points: baseline and post-smoking) as well as cognitive flexibility measurements (one time point: post-smoking).

### 2.3 Materials and measures

#### 2.3.1 Demographic questionnaire

In addition to the FTQ and the questions about the onset and duration of smoking habit, demographic questionnaires included the Alcohol Use Questionnaire (AUQ; Mehrabian and Russell, 1978) and the Drug Use Questionnaire (DUQ; Nesic and Duka, unpublished). The AUQ is a self-report questionnaire which establishes the average weekly alcohol intake and patterns of drinking behavior over a 6-month period. The DUQ is a self-report questionnaire which establishes life-time as well as frequency of current use of illicit drugs, including amphetamines, cocaine, MDMA, opiates, hallucinogens and cannabis. Each of the drugs included in the DUQ is scored as follows: 0 = never used, 1 = not used in the last month, 2 = used once in the last month, 3 = used 2–5 times in the last month, 4 = used 6–10 times in the last month, and 5 = used more than 10 times in the last month (with the exception of cannabis, which is scored as follows: 0 = never smoked, 1 = not smoked in the last month, 2 = smoked once in the last month, 3 = smoked ≤ once a week in the last month, 4 = smoked several times a week in the last month, and 5 = smoked every day in the last month). Analysis is performed on each individual item as well as on the total drug use score which is obtained by adding up all the scores for individual drug items. This DUQ was developed in our laboratory and it has been used for drug screening purposes in a number of studies involving social drinkers (Nesic and Duka, 2006, 2008) and smokers (Nesic et al., unpublished).

#### 2.3.2 Physiological measurements

##### 2.3.2.1 Salivary cotinine level

Each volunteer provided one saliva sample by gently chewing on a cotton swab for 2 min. The swab was then placed in a vial and stored at − 20 °C until analysis. Cotinine measurements were obtained from only 14 LD and 21 HD volunteers since two saliva samples were contaminated with blood and another 11 did not provide a valid measurement. Samples were analyzed using salivary cotinine quantitative enzyme immunoassay kit (Salimetrics). The minimal concentration of cotinine that can be distinguished using this method is .05 ng/ml and the measurements obtained are highly correlated with measurements obtained using liquid chromatography method (r^2^ = .901, p < .001; Dhar, 2004).

##### 2.3.2.2 Breath CO level

Breath carbon-monoxide (CO) level (ppm) was measured using Bedfont Smokerlyzer CO monitor.

#### 2.3.3 Subjective measurements

##### 2.3.3.1 Nicotine-related Visual Analogue Scales (nicotine-VAS)

A list of nicotine-related subjective effects was presented and volunteers were instructed to answer how much each adjective described how they felt at that moment by placing a mark on the bipolar visual analogue scales (VAS — 100 mm) with the poles ‘not at all’ on the left and ‘extremely’ on the right. The adjectives were ‘stimulated’, ‘buzzed’, ‘impatient’, ‘alert’, ‘irritable’, ‘jittery’, ‘dizzy’, ‘relaxed’ and ‘hungrier than usual’ (Jackson et al., 2009 — based on Perkins et al., 1999).

##### 2.3.3.2 Profile of Mood States questionnaire (POMS)

POMS is a list of 72 mood-related adjectives, which are rated on a 5-point scale, ranging from “not at all” [0] to “extremely” [4] (McNair et al., 1971). These items are grouped into 8 basic factors (Anxiety, Depression, Anger, Vigour, Fatigue, Confusion, Friendliness and Elation) as well as two composite scores, Arousal [(Anxiety + Vigour) − (Fatigue + Confusion)] and Positive Mood (Elation − Depression) (de Wit and Doty, 1994).

##### 2.3.3.3 Questionnaire of Smoking Urges (QSU)

The brief version of QSU consists of 10 questions designed to measure desire to smoke and anticipation of positive outcome (factor 1: positive reinforcement) as well as strong urge to smoke and anticipation of relief of withdrawal (factor 2: negative reinforcement) (Cox et al., 2001). The volunteers were required to rate how much each statement applied to them at that particular moment by writing a mark on a Likert-type 7-point scale, ranging from ‘strongly disagree’ (1) to ‘strongly agree’ (7).

#### 2.3.4 Cognitive assessment

##### 2.3.4.1 Intra-Extra Dimensional Set-Shift test (IED)

The IED (Cambridge Neuropsychological Test Automated Battery, Cambridge Cognition) is a computerized touch-screen test of rule acquisition and reversal, which begins as a simple visual discrimination task and then gradually increases in the degree of complexity. The display features two stimuli in the form of shapes and/or shapes and lines that appear randomly in two of the four possible locations on the computer screen. Initially, volunteers are required to learn a simple discrimination (i.e., which of the two shapes is correct), then simple reversal (i.e., change of contingencies, where the previously incorrect shape becomes correct) and then to attend to the correct shape even when the stimuli become more complex by the addition of the lines. Subsequently a new pair of the compound shape-line stimuli appears and volunteers are required to maintain attention to shapes and to ignore the lines (stage 6 — the intra-dimensional [ID] shift). Finally, in stage 8, another new pair of compound stimuli appears and volunteers are now required to switch their attention to the previously irrelevant dimension, the lines (the extra-dimensional [ED] shift). At no point during the test do the participant receive any instructions about the change of rules and thus the learning is entirely feedback-based (signal for ‘right’ or ‘wrong’ at the end of each trial). If a participant makes twenty five errors at any particular stage, the task is terminated prematurely. The main outcome measures derived from this test are the number of stages completed (1–9), total number of errors on the test as well as the number of errors made on each of the nine blocks. Additional two composite outcome measures were analyzed: attentional flexibility (errors in stages 6 + 8) and reversal learning where the same rule applies but the previously incorrect stimulus becomes correct (errors in stages 2 + 5 + 7 + 9).

##### 2.3.4.2 Rapid Visual Information Processing (RVIP) test

A five-minute RVIP test (Jackson et al., 2009 — based on Wesnes and Warburton, 1984) was administered using E-Prime 1.1 software and a response box (Psychology Software Tools Inc). Volunteers were required to monitor a continuous stream of digits, presented at a rate of 80 digits per minute, and to press a response button whenever they saw either three consecutive odd or three consecutive even digits. There were eight such target strings of digits in each one-minute block. The number of correct detections of targets (‘hits’) was recorded within a 1500 ms window following the onset of the third digit in the target sequence. The average latency of correct detections (ms) and the number of false alarms (responses to non-targets) were also recorded. This test was performed four times during the testing session: twice at the beginning of the testing session (practice-a and practice-b runs), once at the pre-smoking baseline (baseline run) and once at the post-smoking time point (post-smoking run).

### 2.4 Experimental procedure

Volunteers reported to the University of Brighton Psychopharmacology Laboratory having been asked to abstain from smoking for a minimum of 2 h (actual abstinence varied between 2 and 96 h, *mean ± SEM*: 12.1 ± 2.4 h). After completing the Smokerlyzer test and providing the saliva sample (volunteers were told that the purpose of both measures was to verify compliance), volunteers were asked to perform the RVIP task twice (practice-a and practice-b runs), with a 10-minute break between the tests, in order to familiarize themselves with the task. Following this and thirty minutes after their arrival to the laboratory, volunteers performed the Smokerlyzer test again followed by the baseline test battery which consisted of nicotine-VAS, QSU, POMS and RVIP (time point: baseline). The experimenter then accompanied the volunteers individually to another room where half were instructed to smoke one of their cigarettes and half remained abstinent but spent a similar period of time in the room. Upon returning to the testing room, volunteers performed the Smokerlyzer test and the second test battery consisting of nicotine-VAS, QSU, POMS, RVIP and IED (time point: post-smoke) followed by the demographic questionnaire. Volunteers were then debriefed about the purpose of the study, paid for their participation and were allowed to leave the laboratory.

### 2.5 Data analyses

Demographic data, duration of abstinence, baseline breath CO and salivary cotinine levels of the four experimental groups were analyzed using univariate analysis of variance (ANOVA) with FTQ group (LD vs. HD) and smoking condition (NS vs. S) as between-subjects factors. The effects of the smoking manipulation on subjective measures as well as on breath CO levels were evaluated using repeated-measures ANOVA with FTQ group and smoking condition as the between-subject factors and time point (baseline vs. post-smoking) as the within-subject factor. IED data were analyzed using univariate ANOVA, with FTQ group and smoking condition as between-subject factors. All significant interactions from repeated measures ANOVA were explored using appropriate post-hoc t-tests while significant interactions observed in the univariate ANOVA of IED data were explored using Tukey's HSD test in order to minimize the occurrence of type 1 error resulting from multiple comparisons.

RVIP data were analyzed twice using repeated-measures ANOVA with FTQ group and smoking condition as the between-subject factors and time as the within-subject factor. The first ANOVA was performed in order to evaluate the effects of the smoking manipulation thus the within-subject factor time had only two levels (baseline vs. post-smoke). The second ANOVA of RVIP data was performed post-hoc in order to evaluate the development of a practice effect and thus the within-subject factor time had four levels (practice-a vs. practice-b vs. baseline vs. post-smoking). Since the distribution of response latencies on RVIP usually shows positive skewness, natural log transformation was applied to normalize these data prior to analyses. Where the sphericity assumption of repeated measures ANOVA was violated, Huynh–Feldt correction was applied. Significant main effects and interactions observed in the second ANOVA of RVIP data (four time points) were analyzed using simple contrasts vs. practice-a run. This analysis was chosen instead of a series of t-test as it was deemed to be the most appropriate way to evaluate the occurrence of a practice effect across the four runs.

To further explore the nicotine dependence level of the present experimental sample, a series of linear correlations of baseline physiological and self-report measures was performed.

Holme's correction was applied to the results of the correlations as well the results of ANOVAs on several factors from the same questionnaire, in order to reduce the likelihood of familywise type 1 error. The adjusted significance level (α′) is calculated by dividing the standard significance level (α = .05) by the number of comparisons performed (c): α′ = α / c. We have decided against applying corrections for multiple comparisons when doing post-hoc t-tests and contrast analyses as this was a simple 2 × 2 design (2 × 2 × 2 for the subjective effect and RVIP variables which were measured before and after the smoking manipulation) and such corrections are not recommended for exploration of significant interactions which involves five or less planned post-hoc comparisons (Roberts and Russo, 1999).

All statistical analyses were performed using SPSS 15.0.

## 3 Results

### 3.1 Population characteristics

The four experimental groups were matched with respect to age as well as alcohol and other drug use (FTQ group × smoking condition Fs[1,44] < 3.78, n.s.). A main effect of FTQ group was revealed for several demographic variables, with HD group having higher FTQ scores, smoking more cigarettes per day, being regular smokers for greater number of years, and reporting shorter duration of abstinence than the LD group (Fs[1,44] > 4.65, ps *<* .05). Demographic characteristics of the two FTQ groups are presented in Table 1.

Salivary cotinine levels ranged between 1.07–29.80 ng/ml in the LD group and 1.87–124.96 ng/ml in the HD group. ANOVA revealed a main effect of FTQ group, with HD having higher salivary cotinine levels than LD volunteers (F[1,31] = 5.522, p *<* .05; Table 1). Analysis of baseline CO levels also revealed a main effect of FTQ group, with LD having lower CO levels than HD (F[1,44] = 6.182, p *<* .05; Table 1).

Several significant correlations were observed between number of cigarettes per day, FTQ score, number of years smoking regularly, duration of abstinence, baseline breath CO levels and salivary cotinine. Most correlations remained significant after the Holme's correction (α′ = .0033 and α′ = .0056 for the largest and the smallest significant effect, respectively; Table 2).

In order to control for the potentially confounding influence that the variable duration of abstinence may have had on the experimental outcome measures, time since last cigarette was entered as a covariate in all subsequent ANOVAs. The results of these analyses of covariance (ANCOVAs) will be reported only when they differed from the results of the original ANOVAs, otherwise only the ANOVA results will be reported here.

### 3.2 Physiological effects

#### 3.2.1 Breath CO level

The ANOVA of change in breath CO levels after the smoking manipulation revealed a significant 2-way interaction of smoking condition and time point (F[1,44] = 133.85, p < .001). Post-hoc t-tests revealed that breath CO significantly increased in the S condition (t[23] = − 11.01, p < .001) and declined in the NS condition (t[23] = 4.51, p < .001) so that at the post-smoking time point levels were significantly higher in the S, compared to the NS group (t[46] = − 2.84, p < .01). While breath CO levels remained higher in the HD group throughout the experiment (main effect of FTQ group: F[1,44] = 5.92, p < .05), this main effect was no longer significant in the ANCOVA controlling for the duration of abstinence (F[1,43] = 2.61, p > .10). The 3-way interaction of FTQ group, smoking manipulation and time point did not reach statistical significance (F[1,44] = .24, n.s.), suggesting that the two FTQ groups did not differ in terms of the amount of smoke inhaled during the smoking manipulation. Breath CO levels of the four experimental groups at both time points are presented in Table 3.

#### 3.3 Subjective effects

**Fig. 1:**
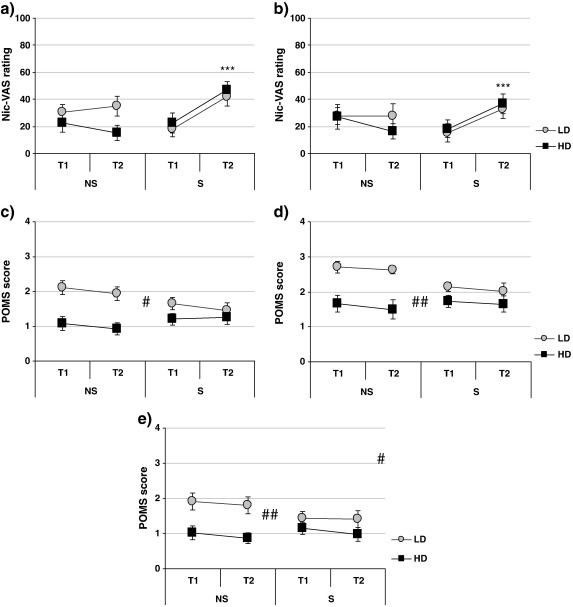
Effects of smoking manipulation (NS-abstinent, S-smoking) on Nic-VAS ratings of a) ‘Buzzed’ and b) ‘Dizzy’ and POMS ratings of c) ‘Vigour’, d) ‘Friendliness’ and e) ’Elation’ in low-dependent [LD] and high-dependent [HD] smokers. Measurements taken at baseline [T1] and after the smoking manipulation [T2]. N = 48. *** p < .001 (T1 vs. T2; paired t-test within S group); ## p < .01, ### p < .001 (main effect of FTQ group, LD vs. HD).

**Fig. 2:**
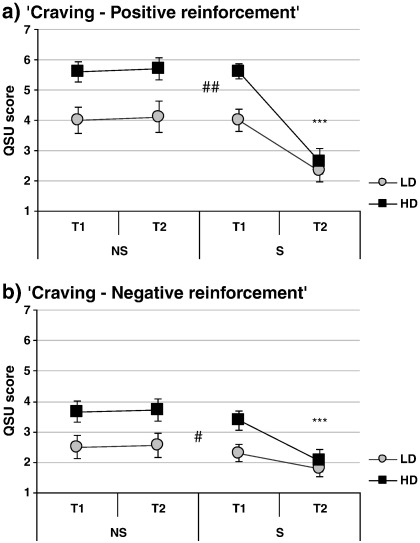
Effects of smoking manipulation (NS-abstinent, S-smoking) on a) QSU factor 1 (positive reinforcement) and b) QSU factor 2 (negative reinforcement) in low-dependent [LD] and high-dependent [HD] smokers. Measurements taken at baseline [T1] and after the smoking manipulation [T2]. N = 48. *** p < .001 (T1 vs. T2; paired t-test within S group); ## p < .01, ### p < .001 (main effect of FTQ group, LD vs. HD).

Interaction of smoking condition and time point was observed for Nic-VAS ratings of ‘Buzzed’ and ‘Dizzy’ (F[1,44] = 13.322, p < .001 and F[1,44] = 9.47, p < .005, respectively — Fig. 1a and b) as well as for both QSU factors (factor 1: F[1,44] = 49.74, p < .001; factor 2: F[1,44] = 14.81, p < .001; Fig. 2). Smoking significantly increased Nic-VAS ratings of ‘Buzzed’ and ‘Dizzy’ (t[23] = − 4.54, p < .001 and t[23] = − 4.28, p < .001, respectively) and decreased QSU scores (factor 1: t[23] = 7.21, p < .001; factor 2: t[23] = 4.11, p < .001), while ratings remained unchanged in the abstinent group (ps > .30).

In addition, several main effects of FTQ group were observed. Compared to LD, HD group had lower scores for POMS factors ‘Vigour’, ‘Friendliness’ and ‘Elation’ (F[1,44] = 14.96, p < .001, F[1,44] = 14.76, p < .001 and F[1,44] = 10.69, p < .005, respectively; Fig. 1c, d and e) as well as higher scores for both QSU factors (factor 1: F[1,44] = 13.23, p < .001; factor 2: F[1,44] = 8.02, p < .01; Fig. 2.).

Three-way interaction of time point, smoking condition and FTQ group did not reach statistical significance for any of the subjective measures (Fs[1,44] < 3.60, ps > .06).

### 3.4 Cognitive effects

#### 3.4.1 IED

The majority of participants (LD: 87.5%, HD: 91.7%) successfully completed all nine stages of the test. ANOVAs of IED variables revealed a significant interaction of FTQ group and smoking condition for the number of the intra-dimensional (ID) set-shift errors (F[1,43] = 11.86, p < .001; Table 4). Post-hoc Tukey's HSD test revealed that HD smokers in the smoking (S) condition made significantly more errors than HD smokers in the abstinent (NS) condition (p < .01). All other comparisons were not significant. Mean values (±SEM) of IED outcome measures of the four experimental groups are presented in Table 4.

Since the number of intra-dimensional errors made by the majority of participants was relatively low, one sample t-tests from 0 were performed in each of the four experimental groups to verify that the observed interaction was unlikely to be a mere statistical artifact due to extreme values. All groups apart from the HD smokers in the abstinent condition made a significant number of errors compared to zero (HD-S: t[11] = 4.75, p < .001; LD-NS: t[11] = 4.69, p < .001; LD-S: t[10] = 2.39, p < .05), confirming the validity of the interaction.

#### 3.4.2 RVIP

Smoking did not significantly modulate any aspect of RVIP performance (Fs[1,44] < 2.65, ps *>* .10). However, when the two practice runs were included in the ANOVA together with the baseline and post-smoke runs, a significant main effect of time was revealed for hits (Huynh–Feldt F[2.64, 116.35] = 28.62, p *<* .001) and false alarms (Huynh-Feldt F[2.08, 91.42] = 9.20, p *<* .001) indicating a steady improvement in RVIP performance across all four runs (simple contrasts vs. practice-a run: Fs > 35.24, ps *<* .001 for hits and Fs > 11.37, ps *<* .005 for false alarms). Response latencies similarly improved and were significantly lower at the post-smoking time point compared to the first practice run (main effect of time: Huynh–Feldt F[2.89, 127.33] = 4.93, p *<* .005; simple contrast post-smoke vs. practice-a run: F[1,44] = 10.12, p *<* .005). Additionally, an interaction of the FTQ group and time point was observed for hits (Huynh–Feldt F[2.64, 116.35] = 3.56, p *<* .05). This interaction reflected a significantly greater rate of improvement in the LD group between the first practice run and the pre-smoking baseline (simple contrast vs. practice-a run: F[1,44] = 8.05, p *<* .01), and this difference was also maintained at the post-smoking time point (simple contrast vs. practice-a run: F[1,44] = 4.36, p *<* .05). Mean values (±SEM) of RVIP variables in LD and HD groups across the four runs are presented in Table 5.

## 4 Discussion

**Fig. 3:**
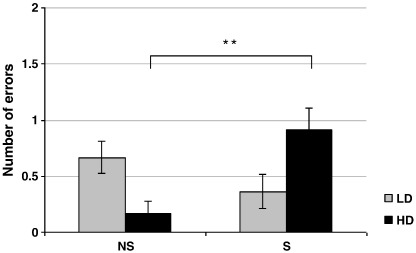
Effects of smoking manipulation (NS-abstinent, S-smoking) on intra-dimensional set-shift performance in low-dependent [LD] and high-dependent [HD] smokers. Measurements taken and after the smoking manipulation [T2]. N = 48. ** p < .01 (unpaired t-test S vs. NS within the HD group).

The present study demonstrated an acute smoking-induced deficit in cognitive flexibility (more errors during the intra-dimensional shift). This effect, however, was not detected in the entire experimental sample but only in individuals with a greater degree of dependence (Fig. 3). Although the higher cotinine (and therefore nicotine) levels inherent in the HD group might have influenced the effect of smoking manipulation on cognitive flexibility, it is clear from the craving data that the HDs, like LDs, were nevertheless in withdrawal and, in fact, their craving in the absence of smoking was higher than that observed in the LD smokers. It could therefore be speculated that smoking is of such an incentive salience for the HD smokers (because of their high dependence) that this reduces the attentional recourses required for the cognitive flexibility.

The finding of smoking-induced deterioration in cognitive flexibility in more dependent smokers contrasts with the previous studies which did not detect an effect of smoking or nicotine patch on cognitive flexibility in samples of heavy smokers (Rotheram-Fuller et al., 2004; Mancuso et al., 1999). However, although the participants in the Rotheram-Fuller et al. study tended to smoke more heavily (> 20 cigarettes per day) than the HD smokers in the present study, they were abstinent only for 30 min, which may have reduced their vulnerability to further smoking-induced deterioration of cognitive flexibility. Thus longer abstinence (minimum two hours in the present study) may be crucial in order to detect acute effects of smoking on cognitive flexibility in high-dependent smokers. In addition, the present study has a number of advantages over the previous ones. In comparison to the Mancuso et al. (1999) study, this study involved a more specific measure of attentional set-shifting as well as a more rapid route of nicotine administration (smoking, as opposed to a nicotine patch). Furthermore, Rotheram-Fuller et al. (2004) study included only 19 participants in their non-opiate-dependent group of smokers and thus had lower power than the present study to detect the effects of smoking on cognitive flexibility.

The lack of significant difference in cognitive flexibility between abstinent HD and LD smokers is not in line with the results of previous correlational studies (heavier smokers display lower cognitive flexibility; e.g. Martin et al., 2000; Kalmijn et al., 2002) as well as studies of abstinent drug users (reduced cognitive flexibility compared to non-drug using controls; Verdejo-Garcia et al., 2004, 2005, 2006; Kim et al., 2005; von Geusau et al., 2004; Ornstein et al., 2000). It is not clear, however, why the smoking-induced impairment occurred selectively in HD but not in LD smokers and future neuroimaging studies should investigate the effect of smoking on brain activation of LD and HD smokers during the performance of a cognitive flexibility test in order to further elucidate the present findings.

It is important to note that the effects of smoking dependence level and of the acute smoking manipulation were observed only for the intra-dimensional set-shift and not for the extra-dimensional set-shift, which is generally more difficult to perform as it requires participants to overcome their acquired bias in order to attend to the previously ignored dimension (Rogers et al., 2000). Successfully performing an intra-dimensional shift is a reflection of whether an individual was able to develop attentional bias for the reinforced dimension by generalizing a discrimination learned for a particular set of stimuli to another set from the same dimension. While schizophrenic patients (Pantelis et al., 1999), heroin addicts (Ornstein et al., 2000) as well as binge drinkers (Scaife and Duka, 2009) have shown selective impairment in intra-dimensional set-shift ability, neuropsychological as well as neuroimaging studies suggest that this ability does not depend solely on pre-frontal cortical activation (e.g. Rogers et al., 2000; Owen et al., 1991; Pantelis et al., 1999). Future studies need to focus on clarifying the origin of this specific deficit as well as its’ relevance to the phenomenon of smoking dependence.

The RVIP test was included in the present study as a control cognitive task known to be sensitive to the effects of nicotine. The lack of the effect of smoking on RVIP performance in the present study is thus in contrast with other studies which demonstrated sensitivity of this test to nicotine manipulations (Warburton and Arnall, 1994; Foulds et al., 1996; Jackson et al., 2009) although negative results using this test have also been reported (Herbert et al., 2001). There was, however, a steady improvement in RVIP performance over the course of the present study (progressive increase in the number of hits and a decrease in the number of false alarms as well as a progressive reduction in response latencies) and it is possible that the effects of smoking in one or both of the nicotine dependence groups would have become apparent if the RVIP test had not been over-practiced over such a short period of time. It is interesting to note that the rate of improvement in RVIP test performance across the four trials was reduced in HD smokers. Since continuous performance tests such as RVIP involve an element of executive control (maintaining and updating information in the working memory and inhibition of processing of and responding to non-target stimuli; see Clark and Goodwin, 2004, for discussion) which can be improved by training (e.g. Persson and Reuter-Lorenz, 2008; Chen et al., 1998), the present finding may suggest that heavy smoking reduces the plasticity which is thought to underlie such practice effects (Westerberg and Klingberg, 2007). This finding thus extends the previous report of dose- and duration-related smoking-induced memory impairment in young smokers (Fried et al., 2006).

In both groups of smokers in the present study, smoking induced an increase in subjective ratings of ‘Buzzed’ and ‘Dizzy’ as well as a decrease in the positive and the negative reinforcement aspects of craving for cigarettes. However, throughout the experimental session, less dependent smokers tended to report less craving for cigarettes compared to the more heavily dependent smokers as well as to feel more vigorous, friendly and elated. Greater vigor has been related to better cognitive performance measured by mental arithmetics and logical memory (Deary and Tait, 1987) and it is thus possible that the increased vigor seen in LD smokers is related to their lower susceptibility to smoking-induced impairment in cognitive flexibility. The present findings of mood differences, together with the finding of reduced practice effect on RVIP in HD smokers, suggest that LD and HD smokers are indeed two distinct populations. This emphasizes the need for separating low- from high-dependent smokers in experimental studies of cognitive function.

As personality traits constitute susceptibility to smoking dependence (e.g. Lipkus et al., 1994), it would be interesting for future studies to examine in more depth the personality characteristics of the two populations of smokers (LD and HD) as this may hold the key both to the difference in their susceptibility to smoking dependence and to the effects of acute smoking on their cognitive function. For instance, a positron-emission tomography (PET) study by Fallon et al. (2004) demonstrated that application of a 21-mg nicotine patch produces no changes in brain activation in low-hostile smokers but induces a widespread decrease in cortical activation in high-hostile smokers. As high hostility is thought to be one of the personality traits associated with susceptibility to nicotine addiction (Lipkus et al., 1994), it would be important to evaluate whether this personality factor also mediates the cognitive effects of smoking on cognitive flexibility.

The difference between LD and HD groups in the number of hours since they last smoked was not related to any of the cognitive or the subjective effect differences between these two groups (inclusion of the duration of abstinence as a covariate did not alter the results of the analyses). This suggests that the present findings are likely to be due to trait and/or chronic nicotine exposure-induced differences between the more dependent and the less dependent smokers and not related to the recent level of nicotine exposure. Although it is possible that higher circulating levels of nicotine in HD smokers (indicated by higher breath CO and cotinine levels) may have contributed to the subjective and cognitive effects observed in the present study, this is likely to be an inherent part of the division between the LD and HD smokers (LD smokers chose to abstain for longer) and it was thus deemed unnecessary to include these variables as covariates in the analyses (see Miller and Chapman, 2001 for a discussion of inappropriateness of covarying for differences which are inherent in group membership).

One of the limitations of the present study is that the between-subjects design employed in the present study does not allow a conclusive comparison of individuals' performance under abstinent and satiated conditions and further studies, employing a within-subjects design, are needed to replicate and elucidate this result. Furthermore, a study comparing abstinent and satiated LD and HD smokers to a control group of non-smokers is necessary to investigate whether the effect of acute and chronic tobacco use on cognitive flexibility is indeed linear and varies directly with the degree of dependence. Inclusion of a smoking control procedure administered in a double-blind manner would also help elucidate the findings from the present study. However, the aim of the present study was to evaluate the cognitive and subjective effects of smoking (and this includes the pharmacological effects of nicotine together with conditioned responses and expectancies) in two populations of smokers who differ with respect to their need for (i.e. dependence on) smoking. Therefore the sham smoking or the denicotinized smoking procedures would be the only options yet these also fall short as adequate controls for smoking in a smoking population.

A further limitation is that IQ was not directly assessed in the present study and, although all participants came from a fairly homogenous population of University students and employees, the possibility of IQ differences between the groups cannot be excluded as a factor contributing to the observed effects. Future studies of cognitive flexibility in smokers should thus control for participants' IQ as a source of variance. Finally, considering the sensitivity of PFC-dependent cognitive functions to circulating levels of sex-related hormones (e.g. Hatta and Nagaya, 2009), another limitation of the present study is that the phase of menstrual cycle of female participants was not controlled for. In order to minimize the hormone-related variability of the data, future studies should include female participants only during the early follicular phase (days 2–4) of the menstrual cycle or those who are taking oral contraceptives.

In summary, influence of smoking on cognitive flexibility varies according to the degree of dependence. In abstinence, HD smokers appear to have greater cognitive flexibility than LD smokers. However, this difference was reversed by smoking, which selectively impaired cognitive flexibility of HD smokers.

It is not clear whether the difference between the LD and the HD smokers in their sensitivity to the smoking-induced impairment of cognitive flexibility is the cause or the consequence of their differential level of dependence. However, the present study demonstrates that the variability in nicotine-dependence levels within the experimental sample may obscure any effects of acute smoking which may be apparent only in the more dependent smokers and only after several hours of abstinence. Dependence levels should therefore be taken into account as a factor in studies assessing the acute as well as chronic cognitive effects of smoking.

## Figures and Tables

**Table 1:** Demographic characteristics and salivary cotinine levels of the two nicotine dependence groups, based on the Fagerström Tolerance Questionnaire (FTQ) score. Values represent means (±SEM). DUQ scoring: 0 = never used, 1 = not used in the last month, 2 = used once in the last month, 3 = used 2–5 times in the last month, 4 = used 6–10 times in the last month, 5 = used more than 10 times in the last month.

n = 24 per group (12 male, 12 female)	Low-dependent smokers [LD]	High-dependent smokers [HD]	t[46]
Age (years)	24.0 (0.7)	22.6 (0.5)	1.68
No. of cigarettes/day	6.8 (0.9)	16.2 (1.4)	−5.76⁎⁎⁎
FTQ score	3.4 (0.2)	6.0 (0.3)	−8.52⁎⁎⁎
No. of years smoking regularly	5.5 (0.6)	7.1 (0.5)	−2.15⁎
Time since last cigarette (h)	18.1 (4.4)	6.2 (1.0)	2.62⁎
Salivary cotinine at baseline (ng/ml)†	9.5 (2.6)	32.0 (8.0)	−2.67⁎
Alcohol use (units/week)	30.0 (3.6)	37.4 (6.8)	−.96
DUQ — Amphetamines	0.3 (0.1)	0.6 (0.2)	−1.34
DUQ — Cocaine	0.5 (0.2)	0.7 (0.2)	−.99
DUQ — Ecstasy (MDMA)	0.5 (0.1)	0.9 (0.2)	−1.47
DUQ — Hallucinogen	0.3 (0.1)	0.6 (0.1)	−1.94
DUQ — Opiate	0.1 (0.1)	0.1 (0.1)	.51
DUQ — Cannabis^a^	1.8 (0.2)	2.1 (0.3)	−.70

a DUQ scoring for cannabis: 0 = never smoked, 1 = not smoked in the last month, 2 = smoked once in the last month, 3 = smoked ≤ once a week in the last month, 4 = smoked several times a week in the last month, 5 = smoked every day in the last month.

⁎ p < .05.

⁎⁎⁎ p < .001 (independent t-tests).

† n = 14 LD, 21 HD.

**Table 2:** Correlations between self-report and physiological measures of nicotine dependence and abstinence. Values represent Pearson's *r.*

*n =* 48	No. of cigarettes/day	FTQ score	No. of years smoking regularly	Time since last cigarette (h)	Breath CO (ppm)	Salivary cotinine (ng/ml)
No. of cigarettes/day						
FTQ score	**.77**⁎⁎					
No. of years smoking regularly	.36	**.41**⁎⁎				
Time since last cigarette (h)	**-.39**⁎⁎	-.29	-.01			
Breath CO (ppm)	**.57**⁎⁎	**.54**⁎⁎	.**45**⁎⁎	-.37		
Salivary cotinine (ng/ml)†	**.50**⁎⁎	**.65**⁎⁎	.28	-.32	**.68**⁎⁎	

**p < .01 (significant after Holme's correction). † n = 35.

**Table 3:** Breath CO levels (ppm) of low- [LD] and high-dependent smokers [HD] allocated to the smoking [S] or the abstinent [NS] condition. Measurements were taken at baseline [T1] and after the smoking manipulation [T2]. Values represent means (±SEM).

*n =* 12 per group (6 m, 6f)		T1#	T2#	
S	LD	2.8 (1.2)	6.7 (1.4)	⁎⁎⁎ ⁎⁎
	HD	8.3 (1.7)	12.3 (1.8)	
NS	LD	5.2 (1.4)	4.4 (1.3)	⁎⁎⁎
	HD	6.8 (1.5)	5.7 (1.2)	

# p < .05 (main effect of the nicotine dependence group – LD vs. HD).

⁎⁎⁎ p < .001 (independent t-tests vs. T1).

⁎⁎ p < .01 (independent t-test vs. NS condition).

**Table 4:** IED test performance of low- [LD] and high-dependent smokers [HD] allocated to the smoking [S] or the abstinent [NS] condition. Test was performed after the smoking manipulation. Values represent means (±SEM).

*n =* 12 per group (6 m, 6f)		NS	S
Number of stages completed	LD	8.8 (0.2)	8.6 (0.4)
	HD	9.0 (0)	8.7 (0.2)
Total number of errors (adjusted for the stages not completed)	LD	17.8 (4.0)	23.3 (10.1)
	HD	11.7 (1.6)	22.3 (5.2)
Number of errors in stage 1 (discrimination learning)	LD	0.2 (0.1)	0.2 (0.2)
	HD	0.0 (0.0)	0.4 (0.2)
Number of errors in stage 2 (simple reversal learning)	LD	1.4 (0.2)	1.2 (0.1)
	HD	1.5 (0.4)	1.2 (0.1)
Number of errors in stage 3 (new dimension introduced but ignored)	LD	1.8 (0.9)	2.1 (0.9)
	HD	1.4 (0.3)	4.2 (1.9)
Number of errors in stage 4 (new dimension still ignored)	LD	0.6 (0.3)	0.3 (0.2)
	HD	0.3 (0.1)	0.5 (0.2)
Number of errors in stage 5 (reversal, still ignoring the new dimension)	LD	1.3 (0.1)	3.1 (2.0)
	HD	1.3 (0.2)	1.1 (0.2)
Number of errors in stage 6 (intra-dimensional set-shift)	LD	0.7 (0.1)	0.4 (0.2)†
	HD	0.2 (0.1)	0.9 (0.2)⁎⁎
Number of errors in stage 7 (reversal, still ignoring the new dimension)	LD	1.4 (0.3)	1.1 (0.1)†
	HD	1.6 (0.4)	1.3 (0.2)
Number of errors in stage 8 (extra-dimensional set-shift)	LD	5.3 (1.9)	4.5 (1.3)†
	HD	4.3 (1.6)	7.0 (2.7)
Number of errors in stage 9 (reversal, still attending to the new dimension)	LD	3.5 (2.0) †	3.1 (1.8)†
	HD	1.2 (0.1)	1.9 (0.6)††
Reversal learning (errors in stages 2 + 5 + 7 + 9)	LD	7.6 (2.2) †	6.4 (1.7)†
	HD	5.6 (0.5)	5.5 (0.6)††
Attentional flexibility (errors in stages 6 + 8)	LD	5.9 (1.8)	4.8 (1.2)†
	HD	4.4 (1.6)	7.9 (2.6)

† N=11.

†† N=10.

⁎⁎ p < .01 (Tukey's HSD test vs. HD-NS group).

**Table 5:** RVIP hits, false alarms and response latencies of low- [LD] and high-dependent smokers [HD] at practice-a run 1 [P-a], practice-b run [P-b], pre-smoking baseline [T1] and after the smoking manipulation [T2]. Values represent means (±SEM). Main effects of time point: ** p < .01, *** p < .001 — simple contrast vs. P1. Time point × FTQ group interaction: # p < .05, ## p < .01 — simple contrast vs. P1.

*n =* 24 per group (12 m, 12f)	FTQ group	P-a	P-b	T1	T2
RVIP — number of hits (max. 40) *** (P2, T1, T2)	LD	24.5 (1.7)	29.8 (1.7)	32.4 ## (1.1)	32.3 # (1.1)
	HD	26.5 (1.7)	29.1 (1.6)	29.9 (1.9)	30.5 (1.9)
RVIP — number of false alarms *** (P2), ** (T1, T2)	LD	3.3 (0.8)	1.8 (0.4)	1.7 (0.4)	1.2 (0.2)
	HD	2.9 (0.8)	1.3 (0.4)	1.4 (0.4)	1.5 (0.6)
RVIP — response latencies (ms) ** (T2)	LD	537.2 (14.9)	526.0 (17.6)	521.6 (15.9)	500.2 (16.9)
	HD	508.2 (15.0)	497.8 (17.4)	505.9 (17.0)	482.1 (16.5)
